# Membrane Topology and Cellular Dynamics of Foot-and-Mouth Disease Virus 3A Protein

**DOI:** 10.1371/journal.pone.0106685

**Published:** 2014-10-02

**Authors:** Mónica González-Magaldi, Miguel A. Martín-Acebes, Leonor Kremer, Francisco Sobrino

**Affiliations:** 1 Centro de Biología Molecular Severo Ochoa, Consejo Superior de Investigaciones Científicas-Universidad Autónoma de Madrid, Madrid, Spain; 2 Centro Nacional de Biotecnología, Consejo Superior de Investigaciones Científicas, Madrid, Spain; Wuhan Bioengineering Institute, China

## Abstract

Foot-and-mouth disease virus non-structural protein 3A plays important roles in virus replication, virulence and host-range; nevertheless little is known on the interactions that this protein can establish with different cell components. In this work, we have performed *in vivo* dynamic studies from cells transiently expressing the green fluorescent protein (GFP) fused to the complete 3A (GFP3A) and versions including different 3A mutations. The results revealed the presence of a mobile fraction of GFP3A, which was found increased in most of the mutants analyzed, and the location of 3A in a continuous compartment in the cytoplasm. A dual behavior was also observed for GFP3A upon cell fractionation, being the protein equally recovered from the cytosolic and membrane fractions, a ratio that was also observed when the insoluble fraction was further fractioned, even in the presence of detergent. Similar results were observed in the fractionation of GFP3ABBB, a 3A protein precursor required for initiating RNA replication. A nonintegral membrane protein topology of FMDV 3A was supported by the lack of glycosylation of versions of 3A in which each of the protein termini was fused to a glycosylation acceptor tag, as well as by their accessibility to degradation by proteases. According to this model 3A would interact with membranes through its central hydrophobic region exposing its N- and C- termini to the cytosol, where interactions between viral and cellular proteins required for virus replication are expected to occur.

## Introduction

Foot-and-mouth disease virus (FMDV) is an aphthovirus that belongs to the *Picornaviridae* family and the etiological agent of an extremely contagious disease of cloven-hoofed animals (FMD) that is responsible for high economic losses in affected countries [1], [2]. FMDV RNA is a positive strand molecule of about 8500 nucleotides that encodes a single ORF [3]. The polyprotein resulting from its translation is processed by viral proteases to yield structural proteins as well as precursors and mature non-structural (NS) proteins [4]. The NS protein 3A is produced by cleavage of 3ABC precursor, reviewed in [5], and is one of the most variable viral proteins encoded by FMDV, being the variable residues preferentially accumulated at its C-terminus [6]. An 18 amino acids long hydrophobic region (HR, spanning residues 59 to 76) is predicted in the middle of the molecule [7], [8], [9]. In other picornaviruses this hydrophobic domain has been reported to target 3A to intracellular membranes [10], [11] and could contribute to locate the viral replication complex within a membrane context [12], [13], [14], [15], but the origin of the membranes involved in FMDV replication and the type of interactions they establish with viral proteins remain uncertain [16]. In cells transiently expressing FMDV 3A, about 50% of the cellular pool of the protein was recovered from the membrane fraction, suggesting an association of 3A with cellular membranes [8].

FMDV 3ABC region shows unique characteristics among picornaviruses, such as encoding 3 copies of viral genome-bound 3B protein [7], [17] that serves as a primer for RNA replication [18]. The three copies of 3B are required for both optimal replication in cell culture [19] and for virulence in natural hosts [20]. In addition, the C-terminal fragment of FMDV 3A (up to the HR) is considerably longer than those of the other picornaviruses. On the other hand, 3A is not the responsible for blocking the endoplasmic reticulum (ER)-to-Golgi transport of proteins as occurs in poliovirus (PV), being this function carried out by 2B and 2BC [8]. FMDV 3A partially colocalizes with ER and Golgi markers [21], [22] and recent evidences point to the involvement of ER exit sites for virus replication, supporting to the involvement of ER in virus replication [23].

On the other hand, 3A protein has been reported to play a role on FMDV host range, as a single amino acid replacement (Q44R) in this protein conferred FMDV the ability to cause vesicular lesions in guinea pigs [24] and deletions and mutations in the C-terminal region associate both to viral attenuation in cattle [25] and to decreased replication rates in bovine epithelial cells [26].

A molecular model of the N-terminal fragment of FMDV 3A protein, derived from the corresponding NMR structure of the PV 3A [27], predicted a hydrophobic interface composed of two α- helices spanning residues 25 to 44 as the main determinant for 3A dimerization. Replacements L38E and L41E, involving charge acquisition at residues predicted to contribute to the hydrophobic interface, reduced dimerization and led to production of infective viruses that replaced the acidic residues introduced (E) by non-polar amino acids, indicating that preservation of the hydrophobic interface is essential for virus replication [9].

To facilitate its study in transient expression assays we fused FMDV 3A wt and mutant versions of this protein − including different deletions, as well as point mutations at the dimerization interface and at the odd cysteine present in 3A − to the green fluorescent protein (GFP). Live cell imaging in combination with photobleaching can provide insights into the movement of proteins and on their interaction with cellular components [28], [29]. Time-lapse microscopy revealed that the cytoplasmic mobility of GFP3A was spatially confined and the analysis of the fluorescence loss in photobleaching (FLIP) [30], [31] supported the location of GFP3A in a continuous compartment in the cytoplasm. In addition, fluorescence recovery after photobleaching (FRAP) analyses [32] revealed the presence of a mobile fraction of GFP3A, which was shown increased in most of the mutants analyzed. On the other hand, biochemical analyses of transfected cells showed that about 60% of GFP3A protein interacted with cellular membranes. Membrane bounded fractions of GFP3A and its precursor GFP3ABBB were further analyzed by different biochemical treatments, resulting in interaction profiles different from that of an integral membrane protein. Further analysis of 3A interactions with membranes by glycosylation tagging experiments and by a biochemical protease protection assay allowed proposing a model of 3A membrane topology.

## Materials and Methods

### Cells and virus

Vero cells (African green monkey epithelial kidney cells; ATCC CCL-81), IBRS-2 (swine kidney cell line) [33], HeLa (human cervical epithelial cells) and BHK-21 cells (Baby hamster kidney cells; ATCC CCL-10) were grown at 37°C and maintained in Dulbecco's modified Eagle's medium (DMEM) (Gibco-BRL), prepared without phenol red for *in vivo* microscopy, supplemented with 5% fetal bovine serum (Gibco-BRL), 2 mM glutamine, 1 µg/ml streptomycin and 1 µg/ml penicillin. A viral stock from type C FMDV C-S8c1 isolate [34] was produced by amplification in BHK-21 cells.

### Antibodies and reagents

Monoclonal Ab (MoAb) 2C2 to NS protein 3A (38), rabbit polyclonal Ab 346 and 479− directed to the C- and N- termini of 3A protein, respectively (9) −, rabbit polyclonal Ab to caveolin-1 (BD Transduction Laboratories), rabbit polyclonal Ab to calreticulin (Abcam) and a MoAb to GFP (Roche) were used.

### Construction of fusion proteins

For *in vivo* experiments 3ABBB, 3A and its mutants (3AL38E, 3AL41E and 3AC65S) were fused to the C-terminus of GFP using plasmid pEGFP-C2 (Clontech). The sequences encoding 3A and 3ABBB wt proteins were amplified by PCR from the infectious clone pMT28 that encodes the genomic RNA of FMDV isolate C-S8c1 [35]. Primers 3A1/3A2 and 3A1/3A-3BBBr were used to amplify 3A wt and 3ABBB, respectively (Table 1). The resulting amplicons and pEGFP were digested with the corresponding restriction enzyme (New England BioLabs), indicated in Table 1, and ligated with DNA ligase T4 (Roche), as described [36]. Substitutions of selected amino acids were generated by site-directed mutagenesis [9]. Deletion mutants were obtained by PCR amplification of the selected 3A sequences: 3AΔHR-C-ter (K53-E153), 3AΔC-ter (R82-E153) and 3AΔN-ter (I1-L41), using primers 3A1/ΔHR-C-ter, ΔC-ter f/ΔC-ter r and ΔN-ter f/ΔN-ter r, respectively. The resulting amplicons were cloned into plasmid pEGFP following digestion with the corresponding restriction enzyme (Table 1). The correct orientation and sequence of the plasmids obtained were confirmed by sequencing with GFP primers.

**Table 1:** Oligonucleotides used for construction of wt and mutant versions of 3A and 3ABBB fused to GFP.

Oligonucleotide	Sequence (5′→3′)	Genomic orientation	Restriction enzyme
3A1	TAGGGGATCCGTATCTCAATACCTTCC	S	BamHI
3A2	GCAGATCTTTATTCAGCTTGCGGTTG	A	BglII
3A-3BBB	GCAGATCTTTACTCAGTGACAATCAA	A	BglII
ΔHR-C-ter	GCAGATCTTTAAAAAGCACGTTTCAC	A	BglII
ΔC-ter f	GCGAAGCTTTCTAGAAATGATCTCAATACCTTCC	S	HindIII
ΔC-ter r	GCCGGATCCTTACTTGTGAGTCTCGC	A	BamHI
ΔN-ter f	CGGAGATCTGGATCCAACAAACTTCA	S	BglII
ΔN-ter r	GCAAGCTTTTATTCAGCTTGCGGTTG	A	HindIII
GlyN-ter F	TTCGCGGATCCGACATGAATTCGACCTCGGCTACATCTCAATACCTTCCCAA	S	BamHI
GlyTMC-ter F	TTCGCGGATCCGACATGAATTCGACCTCGGCTACTTTGAAATTGTTGCACTG	S	BamHI
C-terGly R	TCGCCTCTAGACTAGTTAGCCGAGGTCGAATTTCAGCTTGCGGTTGCTC	A	Xba I

### N-glycosylation insertion mutagenesis and deglycosylation assays

For topologic analyses 3A protein was cloned in pcDNA3.1+ vector (Invitrogen) using 3A wt amplicons and the restriction enzymes BamHI and XbaI. To construct plasmids pcDNA3A-glyc, pcDNAglyc-3A, and pcDNAglyc-3AΔN-ter(I1-N58), a N-glycosylation acceptor site (Asn-Ser-Thr-Ser-Ala-Asn) (36) was fused in-frame to the C- or N-termini of 3A, or to the N-terminus of 3AΔN-ter. Amplicons were obtained by PCR using pcDNA3A as template, the sense primers Gly-Nter F, Gly-TMC-ter F, and the antisense primer C-ter-Gly R with the corresponding sense and antisense primers (Table 1). For the deglycosylation assay, Vero cells were grown in 35-mm dishes, transfected with 2 µg of DNA and 24 h post transfection (pt) lysed in 200 µl NBP [50 mM Tris-HCl, pH 7.5, 150 mM NaCl, 1% NP-40, 1% sodium deoxycholate, 0.1% SDS, 1 mM phenylmethylsulfonylfluoride, protease inhibitor cocktail 1x (Roche)], treated with 1 µl benzonase (Novagen). Lysed cells were centrifuged 10 min at 300×g and the supernatant centrifuged 30 min at 30000×g. Pellets were resuspended in NPB and deglycosylation was performed with PGNase F (New England Biolabs) as recommended by manufacturer. Laemmli sample buffer [37] was added and proteins were separated by SDS-PAGE and analyzed by Western blot. As positive controls pTM-DV-NS4A(1–150)-GFP-Glyc and pTM-DV-NS4A(1–100)-GFP-Glyc − expressing dengue virus NS4A full length (FL) and a C-terminal truncation of NS4A (amino acids 1–100), respectively [38] − were transfected. One h before transfection with these plasmids, cells were infected with vaccinia virus VTF7–3 expressing the T7 RNA polymerase to allow cytoplasmic transcription of the constructs [39]. At 20 h pt cells were lysed and processed as described above.

### Biochemical treatment of cell lysates

Vero cells were grown in 60-mm dishes and transfected with 1–2 µg of plasmid DNA using Lipofectamine (Invitrogen). 24 h pt cells were lysed with PBS supplemented with protease inhibitor cocktail 1x (Roche) by five cycles of freezing in liquid nitrogen and thawing at 37°C. Lysed cells were centrifuged at 300×g for 10 min and then at 30000×g for 20 min twice. Pellets were resuspended in the following solutions: 0.1 M Na_2_CO_3_, 4 M Urea (Merck), 1 M KCl (Merck), or 0.5% Triton X-100 (Sigma). Samples were boiled, resolved on SDS-PAGE and immunoblotted, following addition of Laemmli sample buffer.

### Biochemical protease protection assay

HeLa cells grown in 35-mm dishes, without or with coverslips (in case of immunofluorescence analysis), were transfected with pcDNA3A. This cell line was used because of it high level of transfection efficiency. 24 h pt cells were washed with KHM buffer (110 mM potassium acetate, 20 mM HEPES pH 7.2, 2 mM MgCl_2_) and permeabilized with 50 µM digitonin in KHM buffer for 1 min at room temperature. Then, cells were washed in KHM and treated with 0.025% trypsin or 50 µM proteinase K for 5 min at room temperature. Finally, cells were washed and lysed, Laemmli sample buffer was added, and proteins were separated in a SDS-PAGE and analyzed by Western blotting with antibodies to the N- and C-termini of 3A and to calreticulin. For immunofluorescence analysis, cells in coverslips with the same treatment were fixed in 4% paraformaldehyde.

### Western blot analysis

Vero cells grown on 35-mm dishes were transfected as described above with 1 µg of different plasmids. At 24 h pt, cells were scraped on ice into NP-40 lysis buffer (10 mM EGTA, 2.5 mM MgCl2, 1% NP-40, 20 mM HEPES pH 7.4) and sonicated. Equal volumes of each sample mixed with Laemmli sample buffer were boiled, separated by SDS-PAGE 12%, and transferred onto a nitrocellulose membrane. The membrane was blocked, and proteins were detected by incubation with the selected primary antibody and the corresponding horseradish peroxidase-coupled secondary antibody that was developed using a chemiluminescence kit (Perkin-Elmer).

### Density gradient fractionation

The procedure for isolation of Triton X-100-insoluble membranes by centrifugation to equilibrium in sucrose density gradients was essentially as described [40]. Cells grown in 100-mm dishes were transfected and 24 h later washed tree times with cold PBS, scraped on 0.5 ml of 0.5% Triton X-100 in TNE buffer (25 mM Tris-HCl, 150 mM NaCl, 5 mM EDTA pH 7.4) and maintained for 30 min on ice. The lysate was passed through a 22-gauge needle, mixed with 70% sucrose in TNE buffer supplemented with 1 mM PMSF and protease inhibitor cocktail, and brought to 35% sucrose in a final volume of 4 ml beneath a 8 ml 5–30% linear sucrose gradient. Gradients were centrifuged at 4°C for 18 h at 180000×g in a SW40 rotor (Beckman) and 12 fractions of 1 ml, collected from top to bottom, were analyzed by Western blotting or stored at −80°C.

### Immunofluorescence and confocal microscopy

Cells grown on glass cover slips were transfected or infected with FMDV C-S8c1 (moi  = 5 PFU/ml). At 24 h pt or 4 h post infection (pi), cells were fixed in 4% paraformaldehyde for 15 min at room temperature, blocked, and permeabilized with PBTG buffer (0.1% Triton X-100, 1% bovine serum albumin (BSA), and 1 M glycine in PBS) for 15 min. Samples were incubated with the selected primary antibody diluted in 1% BSA in PBS for 1 h at room temperature, washed with PBS and incubated with the corresponding secondary antibody for 30 min. Finally, samples were mounted in Fluoromount G (Southern Biotech) and cells were observed with a Microradiance confocal (Biorad/Zeiss) microscope.

### Time-lapse microscopy

Time-lapse was performed in Vero cells grown on glass bottom 35-mm dishes (MatTek) transfected with GFP3A, which 6 h pt were transferred to the microscope incubator previously warmed at 37°C. Images (5 different planes along Z axis at intervals of 5 min for 3 h) were acquired using an inverted Axiovert200 microscope (Zeiss) with a 63x/1.2 Water C-Apochromat Corr, coupled to a digital camera C9100–02 (Hamamatsu). Humidity, CO_2_ and temperature (37°C) were controlled using the *In Vivo* Cell Observer system (Zeiss). Manual tracking of the fluorescence was performed using ImageJ plug-in: Manual tracking.

### FRAP analysis

Vero cells grown on glass bottom 35-mm dishes were transfected with GFP3A. At 6 h pt, cells were observed using an *in vivo* system in an inverted Axio Observer Confocal laser scanning microscope (Zeiss), in triplicate experiments (n>10 cells/experiment). The 488-nm laser line was used to perform photobleaching of a defined circular 10 µm ø region of interest (ROI) at full laser power (100% laser power, 100 interactions). Recovery of the fluorescence was monitored by continuous scanning of a control ROI either in a neighbor cell or at a different region in the cytoplasm of the same cell, using a low laser power (1%) until the fluorescence of the bleached area reached a plateau. Cells were scanned six times before photobleaching to determine the maximum initial intensity of fluorescence. No additional photobleaching was observed during recovery. The images were captured with the Zenon (Zeiss) software. Fcalc program, Turu Centre for Biotechnology, Finland [41] was used to analyzed the FRAP data and to calculate the mobile fraction fitting an exponential curve to the corrected data using a least square fit: A1 (1-ek1t) +A2 (1-ek2t), where A1 and A2 represent the mobile fractions with a two function fit, and k the kinetic constant.

### FLIP analysis

Cells grown on glass bottom 35-mm dishes were transfected with pEGFP3A or plgLdR1KDEL-RFP [32]. Twenty four h pt cells were observed as described before. The 488-nm laser line was used to carry out sequential photobleaching events of a selected ROI. This area was exposed to 15 interactions of 100% laser power for RFP and 60% for GFP every six scanners (7 s) for 150 repeats. Loss of fluorescence was monitored in a different ROI in the cytoplasm of the same repetitive bleached cell. Fluorescence intensity of a neighbor cell ROI was determined to estimate global photobleaching in the field. Images were analyzed with the Zenon program.

### Data analysis

To probe statistical significance of the data, one-way analysis of the variance was performed with statistical package SPSS 19.0 (SPSS, Inc.) for Windows. For multiple comparisons, Bonferroni's correction was applied. The data are presented as means ± the standard deviations and statistically significant differences are indicated in the figures by an *.

## Results

### Characterization of 3A fluorescent protein in transfected cells

**Figure 1:**
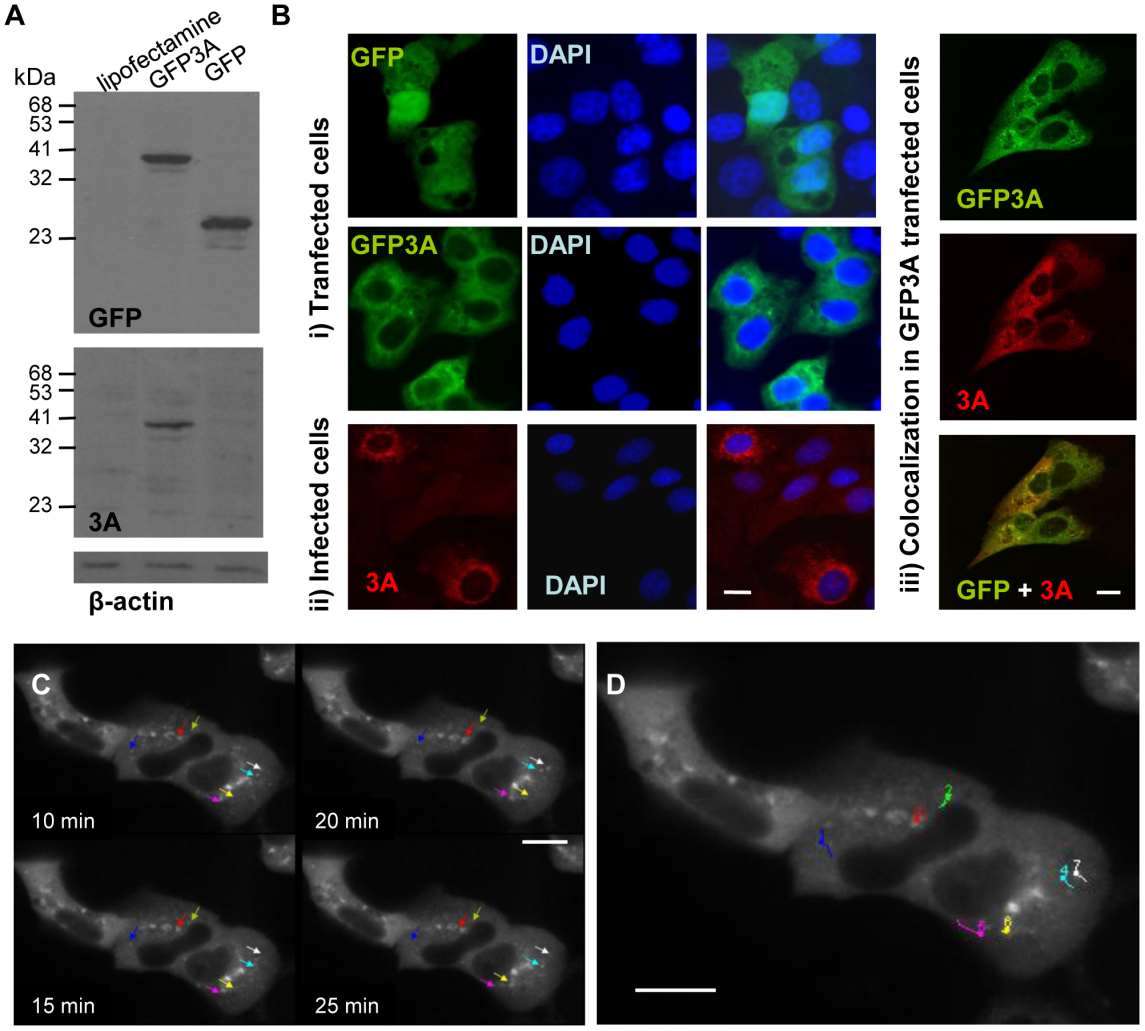
Expression of GFP3A and *in vivo* analyses of 3A protein in transfected cells. A) Vero cells were transfected with 1 µg of pEGFP3A. Proteins were detected by Western blotting with a polyclonal Ab to 3A (479) or a MoAb to GFP as primary antibodies. Blotting to β-actin was used as control of protein loading. Molecular weights are indicated in kDa. B) Fluorescence microscopy of: i) Vero cells transiently expressing GFP or GFP3A; ii) IBRS cells 4 h pi with FMDV; iii) Vero cells transiently expressing GFP3A (24 h pt) incubated with a polyclonal Ab to 3A (346) (red) or showing the autofluorescence of GFP (green). Co-localization is shown in the merge image. Cell nuclei were counterstained with DAPI. C) For time-lapse microscopy, Vero cells were transfected with pEGFP3A and 6 h later cells were scanned by 488-line laser every 5 min for 3 h, as described in Materials and Methods. Images at different times are shown. Colored arrows point to selected tracked dots. D) Manual tracking of the fluorescence of selected dots was performed using ImageJ plug-in. Scale bar, 20 µm.

The analysis of protein dynamics *in vivo* requires fluorescent probes whose biophysical properties can be monitored to infer changes in cellular biochemistry [28], [42]. In this work we have studied *in vivo* the properties of FMDV 3A protein by means of its fusion to GFP. A bioinformatic application (TMHMM) [43] that estimates the likelihood of membrane protein topology indicated that fusion of GFP to the N-terminus, but not to the C-terminus of 3A, maintained the topology of this viral protein. Therefore, GFP was cloned as fusion with the N-terminus of 3Awt, and expression of GFP3A confirmed by Western blot analysis (Fig. 1A). As a first step in the use of GFP3A as a tool to study the 3A *in vivo* distribution and dynamics, transfected cells were examined by confocal microscopy. Fluorescence of GFP and GFP3A in Vero cells is shown in Fig. 1B-i. While GFP fluorescence was observed throughout the whole cell including the nucleus, GFP3A fluorescence was restricted to the cytoplasm, including a perinuclear distribution similar to that found for 3A in FMDV-infected IBRS-2 cells (Fig. 1B-ii). As expected, fluorescence of GFP and 3A were shown to colocalize in transfected cells (Fig. 1B-iii).

Then, the cytoplasmic distribution of GFP3A was analyzed by time-lapse microscopy. Representative images of Vero cells 6 h pt with pEGFP3Awt are shown in Fig.1C. The *in vivo* record showed a pattern of fluorescence with puncta located predominantly in the perinuclear region as well as a diffuse fluorescence dispersed in the cytoplasm. Tracking of punctate structures revealed a confined movement pattern represented in the trajectories drawn in Fig. 1D, and the velocity of the fluorescence puncta ranged from 0.1 to 0.7 µm/s. This confined pattern is different from those described for other viral proteins associated to microtubules that usually move over longer distances [32], [44].

### Cellular dynamics of GFP3A protein

**Figure 2:**
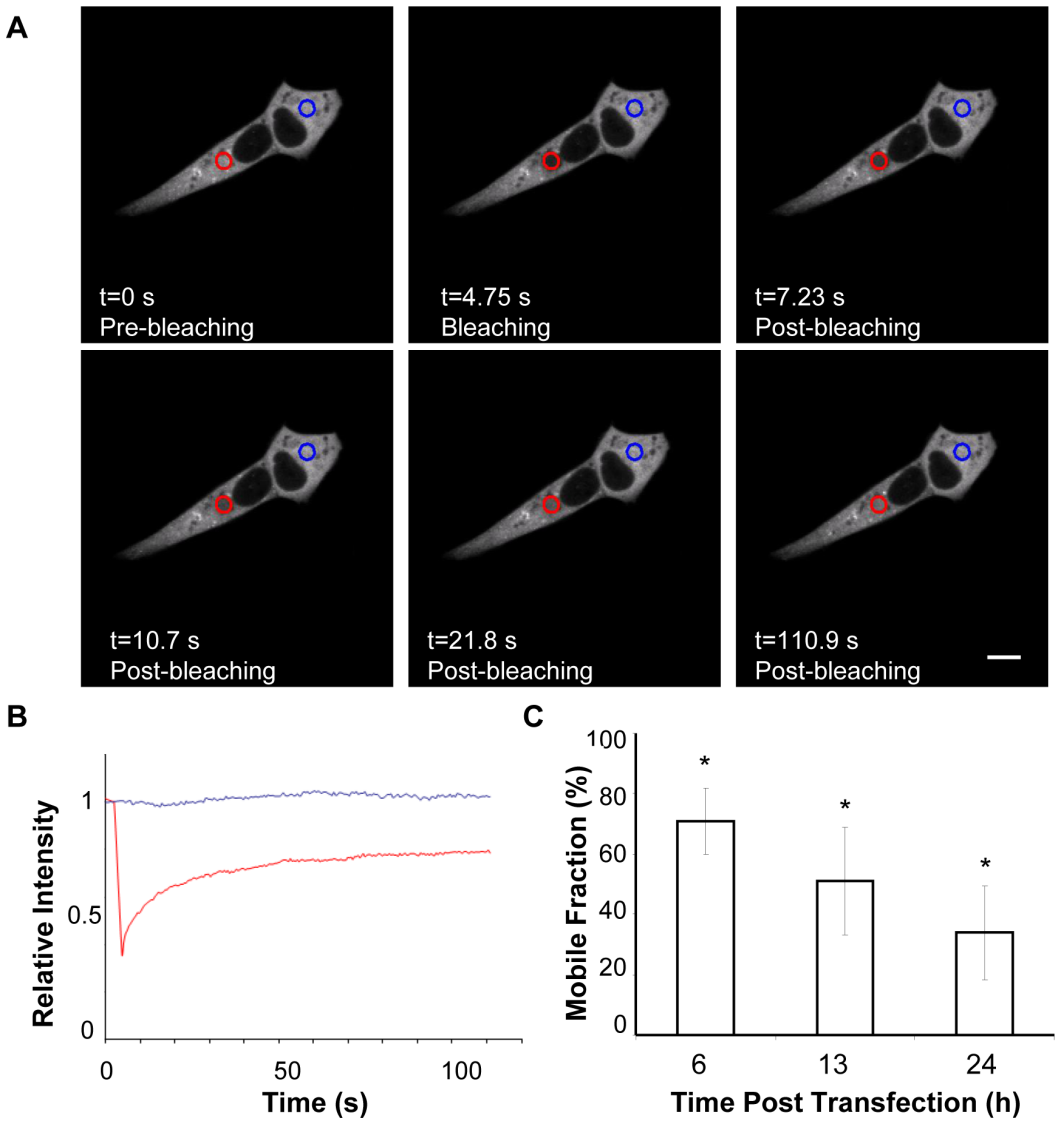
FRAP analysis. Vero cells were transfected with pEGFP3A. A) Images of transfected cells pre bleaching, bleaching and post bleaching. At different times pt a ROI of 10 µm Ø circular region (red circle) was photobleached. Recovery of the fluorescence was monitored by continuous scanning the whole cell (including ROI). The area selected as control in the neighbor cell is indicated by a blue circle. Experiments were done in triplicate (n>10). B) Relative intensity vs. time in FRAP determined in (A). C) Percentages of the GFP3A mobile fraction, determined as described in Materials and Methods, at different times pt. Data are presented as means ± the standard deviations. An asterisk denotes statistically significant differences (P≤0.005). Scale bar, 20 µm.

Time-lapse imaging of proteins with a restricted mobility has to face limitations imposed by long time and repetitive expositions of cells to the excitation light that are detrimental for cellular viability. Consequently, FRAP was used to further investigate the movement of GFP3A in the cytoplasm of cells at different times pt. As shown in Fig. 2, a fraction of GFP3A recovered fluorescence after photobleaching indicating that, when transiently expressed, 3A protein shows a mobile (Mf) and a non- mobile fraction. From the images in Fig. 2A-B a Mf of 35±15% was determined at 24 h pt. Higher Mf values were found at shorter times pt, being of 71±11% at 6 h pt (Fig 2C). Most FRAP experiments are usually conducted between 16 to 36 h pt to ensure the correct expression of the transiently expressed protein, to have enough bright specimens, as well as to minimize overexpression artifacts [45]; for this reason further FRAP analysis were performed at 24 h pt.

**Figure 3:**
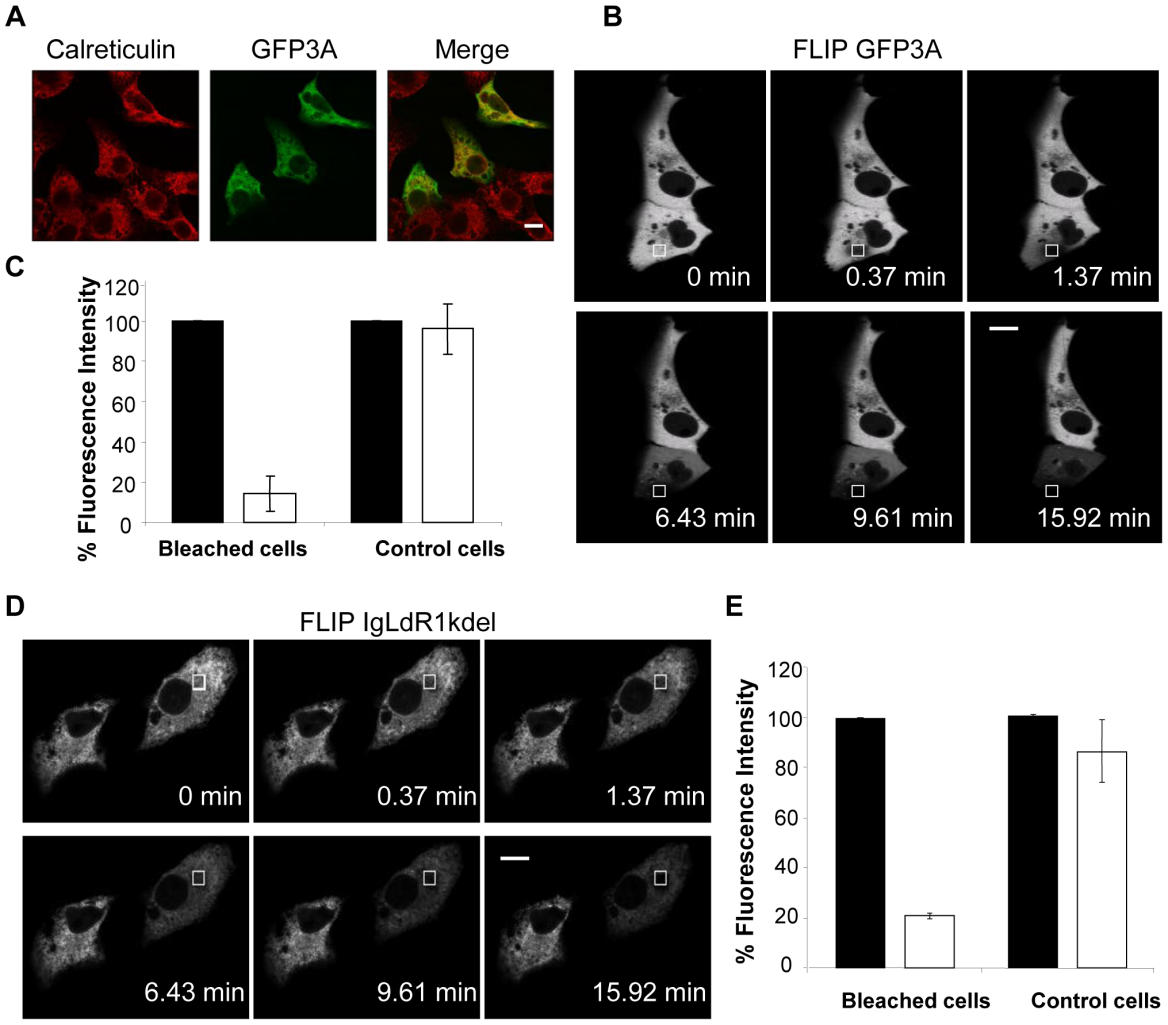
Distribution of GFP3A in the cytoplasm of transfected cells. A) Colocalization of GFP3A and calreticulin in Vero cells transfected with pEGFP3A. B) FLIP analysis; images of pre and post bleached cells. Vero Cells were transfected with pEGFP3A and 24 h pt the indicated area (white rectangle) was subjected to sequential photobleaching. An image of the field was acquired after each bleaching event to determine the loss of fluorescence in the cytoplasm of the cell. C) The percentage of the fluorescence intensity determined in B is represented for bleached and neighbor control (not bleached) cells. Mean fluorescence intensities of prebleached events (black bars) and after all the bleaching repeats (white bars) are indicated. FLIP analysis, as in (B), of cells transfected with plgLdR1KDEL-RFP. E) Percentage of the fluorescence intensity determined in (D). Scale bar, 20 µm.

As commented in the Introduction, FMDV 3A protein has been associated to the ER, so we analysed the localization of GFP3A by confocal microscopy. As reported for transiently expressed 3A [22], our fusion protein colocalized with the ER marker calreticulin (Fig. 3A), confirming the association of GFP3A with this organelle. It is well documented that membranes and luminal spaces of the ER are normally continuous throughout the cell and that rough and smooth ER form an interconnected membrane system [31], [46], [47], which has been confirmed by FLIP [30], [48]. Therefore, we decided to repetitively photobleach a selected fluorescent area in the cytoplasm to measure the fluorescence loss in photobleaching in cells transfected with GFP3A. A mouse IgH leader sequence-derived ER targeted mRFP1 version, termed IgLdR1kdel [32], was used as control of a ER-resident protein. As expected for a protein located at a continuous compartment, repetitive photobleaching of a region in the cytoplasm led to extinction of IgLdR1kdel fluorescence in the whole cell (Fig. 3B–C). A similar behavior was found in cells expressing GFP3A (Fig. 3D–E), supporting that this protein is also placed in a continuous cytoplasmic compartment, which is compatible with its localization at the ER.

### Analysis of different mutants of GFP3A protein

**Figure 4:**
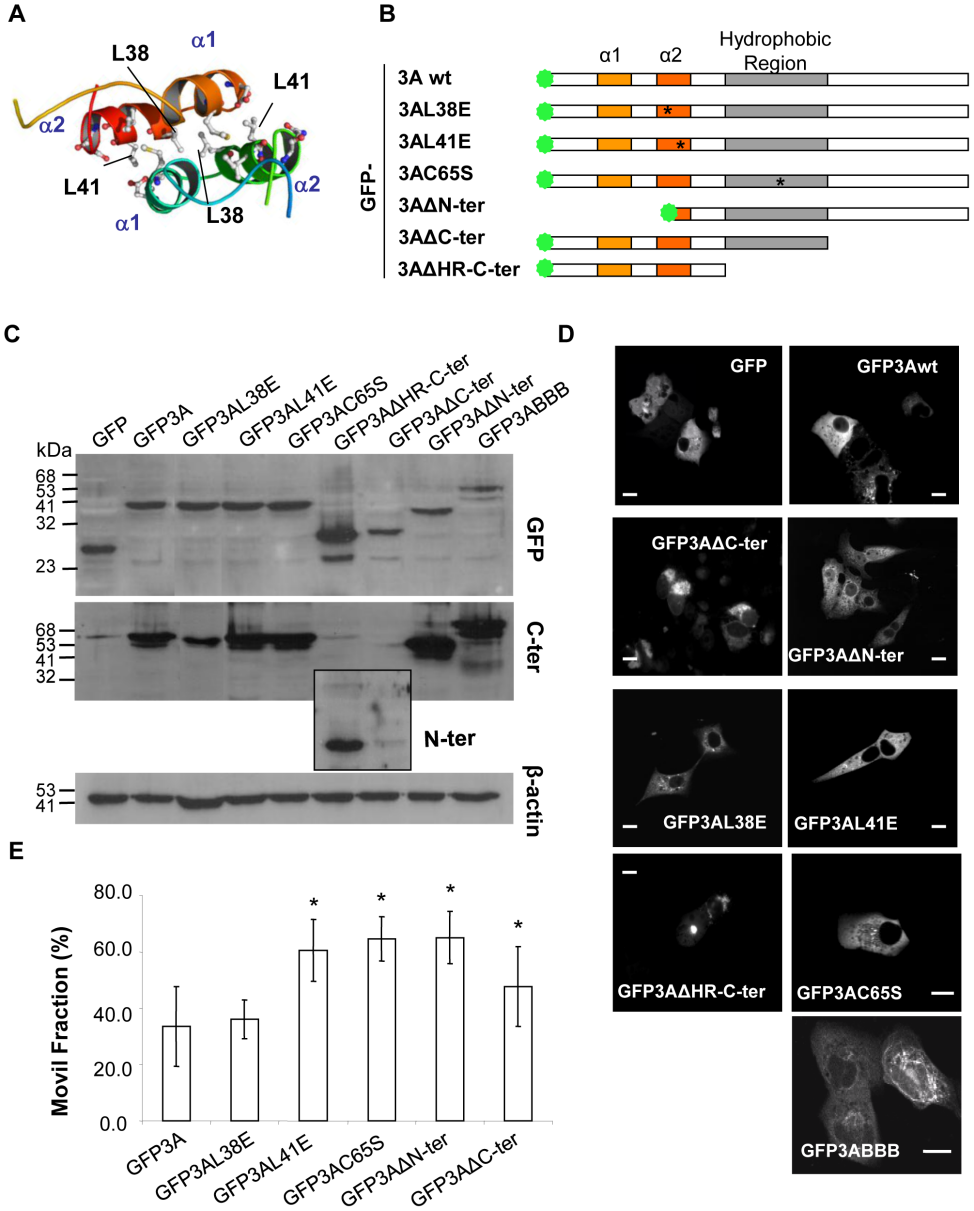
Analysis of GFP fusion proteins carrying mutations in 3A. A) Structural model for FMDV 3A protein dimer (9). Ribbons represent α-helixes 1 and 2. Leucines at positions 38 and 41 are indicated. B). Schematic representation of the fusion proteins analyzed in which GFP (green stars), α-helixes (orange boxes) and the hydrophobic region (gray boxes) are indicated. Substitutions (L38E, L41E and C65S at residues conserved in 99, 99 and 85% among the FMDV sequences from the NCBI database, respectively) and deletions − ΔN-ter (I1-L41), ΔC-ter (R82-E153) and ΔHR-C-ter (K53-E153) −, generated as described in Materials and Methods, are shown. Asterisks denote single replacements. An alignment of the 3A sequences spanning the different mutations constructed among different FMDV serotypes can be found at [9] C) Western blotting of cells transiently expressing fusion proteins. Vero cells were transfected with 1 µg of plasmids expressing the fusion proteins indicated. Proteins were detected by incubation with a primary polyclonal antibody to the C-terminus (346) − with the exception of GFPΔHR-C-ter and GFPΔC-ter (shown boxed) that were blotted with serum 443 to the N-terminus − or with a MoAb to GFP. Blotting to an anti-β-actin was used as control of protein loading. Molecular weights are indicated in kDa. D) Fluorescent pattern of different GFP fusion proteins. Vero cells 24 h pt with the plasmids expressing the fusion proteins indicated were fixed and processed for confocal microscopy as described in Materials and Methods. E) Comparison of mobile fractions in FRAP of GFP3A fusion proteins. Vero cells were transfected as in (D), and 24 h pt FRAP was determined as described in the legend of Fig. 5. Data are presented as means ± the standard deviations of triplicate experiments (n>10). Statistically significant differences relative to GFP3A percentage of mobile fraction are indicated by an asterisk (P≤0.001). Scale bar, 20 µm.

Based on the predicted structure of FMDV 3A [9], different mutations were introduced to analyze their effect on the properties of this protein (Fig. 4A–B). These mutations included: i) replacements L38E and L41E that had been shown to destabilize the hydrophobic interaction between residues involved in 3A dimerization, ii) substitution of the odd cysteine present in 3A and located in the HR (replacement C65S), iii) a truncated protein lacking the N-terminal region of 3A (ΔN-ter), and iv) truncated proteins lacking the C-terminal region and maintaining (ΔC-ter) or not (ΔHR-C-ter) the HR. The 3ABBBwt precursor was also included in this study. All constructs were fused to the C-terminus of GFP, and their expression was confirmed by Western blot analysis as a band of the expected electrophoretic mobility was found for each construction (Fig. 4C). In addition, no major differences in the distribution of the fusion proteins were observed by fluorescence microscopy of Vero cells transfected with each of the plasmids, with the exception of GFP3ABBB whose distribution was similar to that reported for 3ABBB [21], and of GFP3AΔHR-C-ter whose fluorescence was accumulated in the cytoplasm of cells that showed an altered morphology and pyknotic nucleus (Fig. 4D). For this reason GFP3AΔHR-C-ter was not included in the subsequent *in vivo* analysis. The trend of GFP3AΔC-ter to appear accumulated in the cytoplasm and the cell alterations associated to its expression were of lower magnitude that those observed for GFP3AΔHR-C-ter; therefore, the properties of GFP3AΔC-ter were further analyzed.

The different 3A mutants constructed were analyzed in FRAP experiments and their Mfs compared with that of GFP3A (Fig. 4E). With the exception of replacement L38E at the dimerization interface that did not alter the mobile fraction of the protein, the remaining single mutations resulted in a significant increase of Mfs values. Such increase was also observed in the deletion mutants GFP3AΔC-ter and GFP3AΔN-ter analyzed. Thus, different 3A mutations can alter the interactions responsible for the mobility observed for GFP3A.

**Figure 5:**
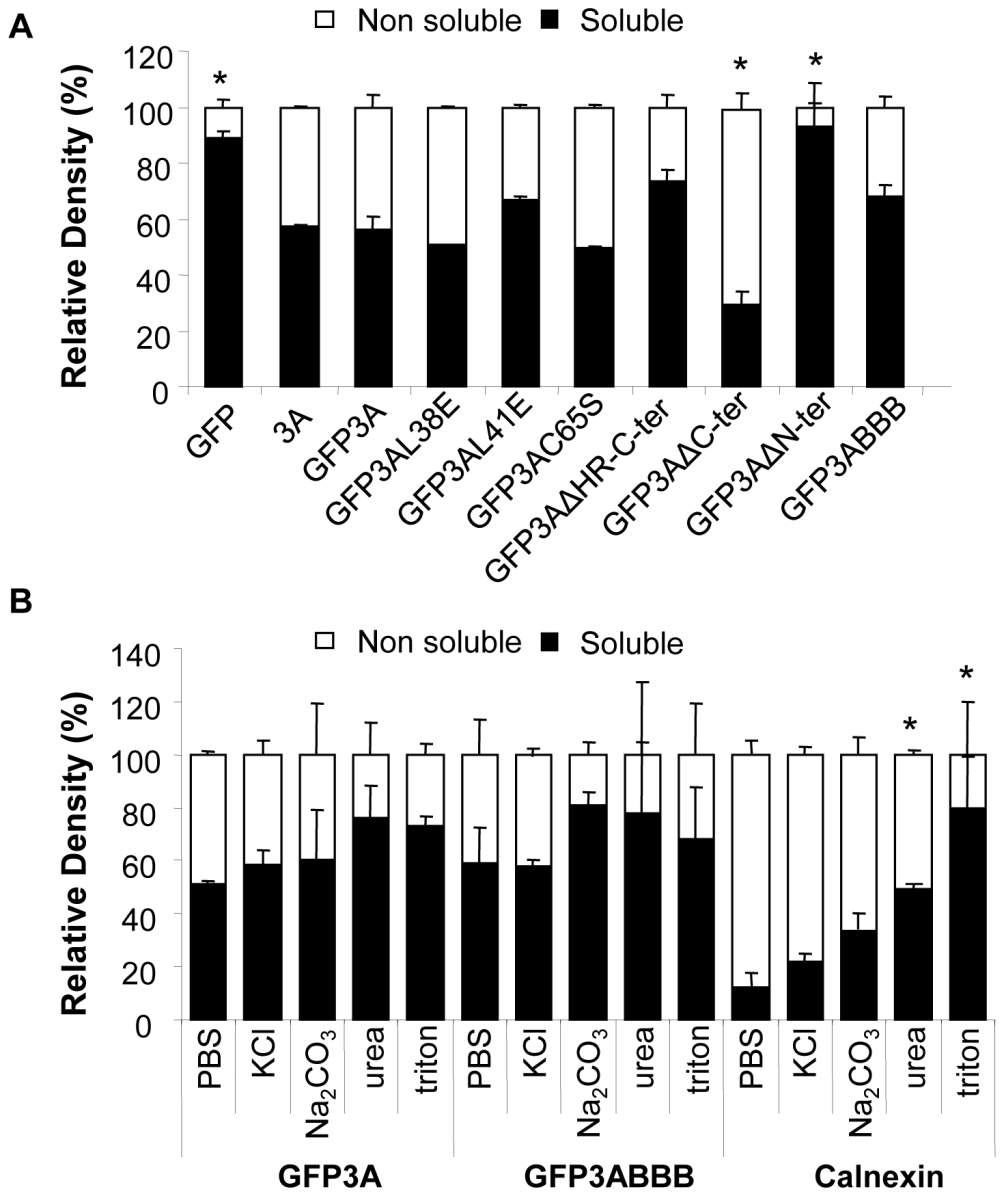
Solubility of GFP3A fusion proteins. A) Distribution of fusion proteins in soluble or insoluble fractions of transfected cells. Vero cells transfected with 1 µg of pEGFP, pRSV3A and the plasmids expressing the fusion proteins indicated, were lysed in PBS buffer by freeze–thawing and fractionated by centrifugation. Proteins in pellets and supernatants were resolved on a 12% SDS-PAGE, transferred to a membrane, and blotted with MoAb to GFP or 3A (2C2). Statistically significant differences, relative to GFP3A are indicated by * (P≤0.05). B) Insoluble fraction association of transiently expressed GFP3A and GFP3ABBB proteins. Vero cells transfected with 1 µg of pEGFP3A or pEGFP3ABBB, were processed as in (A). Pellets were further treated with: Na_2_CO_3_, Urea, KCl or Triton X-100 (as described in Materials and Methods) and their proteins blotted with MoAb to GFP or to calnexin. Plots represent the percentage of the relative intensity of the protein bands in the blot that were quantified by densitometry with ImageJ program. Statistically significant differences, relative to PBS treatment, are indicated by * (P≤0.05).

To gain insight on the interactions established by 3A with cellular membranes, biochemical analyses were performed with cells transiently expressing the 3A fusion proteins. The solubility of the GFP3A, GFP3ABBB and the mutant fusion proteins was analyzed from supernatants (soluble fraction) and pellets (membrane-associated insoluble fraction) recovered after centrifugation of lysates from transfected cells. The presence of 3A was revealed by immunoblotting using MoAb to 3A and to GFP. In Fig. 5A the relative percentage of the Ab staining intensity in the soluble and the insoluble fractions is represented for each fusion protein; in these analyses a non-fused 3A wt protein (pRSV3A), as well as GFP were included. A similar proportion, about 60%, of the cellular pools of FMDV GFP3A and 3A was detected in the soluble fraction while the remaining protein was found in the membrane fraction supporting the partial association of both proteins with intracellular membranes. As observed with the substitution of the odd cysteine C65S, replacements 3AL38E and 3AL41E that diminish 3A dimerization, did not alter the protein solubility. In the case of the precursor GFP3ABBB a slightly increase was observed in the proportion of protein recovered in the soluble fraction, although values were not statistically significant. Among the deletion mutants, GFP3AΔC-ter showed the highest decrease in solubility, while GFP3AΔN-ter, the 3A version with the N-terminus truncated in the predicted dimerization region, was the most soluble of the proteins analyzed. These results suggest that N-ter contributes to 3A insolubility although the single replacements that impair dimerization did not significantly affect the solubility of this protein.

### Biochemical characterization of the interaction of 3A with cell membranes

Peripheral and integral membrane proteins differentially respond to treatments with high salt, high pH, or chaotropic reagents such as guanidine or urea that will dissociate peripheral membrane proteins from the lipid bilayer [49], [50]. In contrast, interaction of integral membrane proteins with the lipid bilayer is much stronger than that of peripheral membrane proteins, requiring the use of detergents for its membrane extraction [15].

Given that about half of 3A was found associated to membranes in transfected cells, the subcellular membrane fractions including GFP3A and its precursor GFP3ABBB were subjected to further biochemical treatments to characterize its interactions with cell membranes (Fig. 5B). Pellets of lysed transfected cells were either dissolved in PBS or treated with mild chaotropic salt conditions (4 M urea), high pH (0.1 M Na_2_CO_3_) or high salt concentration (1 M KCl), as described [11], [51], as well as with a non-ionic detergent (0.5% Triton-X 100), prior to a second fractionation by centrifugation. The effect of these treatments on calnexin was analyzed, as a control for the behavior of an integral membrane protein. As expected statistically significant increases in calnexin solubility were only observed upon treatment of pellets with triton X-100. The solubility of GFP3A and GFP3ABBB differed from that of calnexin. Upon centrifugation the solubility in PBS of GFP3A and GFP3ABBB, was of about 50%, a value similar to that obtain for 3A and GFP3A in the first fractionation, suggesting a dynamic equilibrium between membrane-associated and soluble GFP3A that pulls protein from the membrane when the initial soluble protein is removed. Furthermore, no statistically significant increases in solubility were observed with any of the treatment conditions tested. These results indicate that 3A and its precursor 3ABBB interacts with cellular membranes in a manner different from that of an integral membrane protein.

### Lack of interaction of 3A with lipid rafts

**Figure 6:**
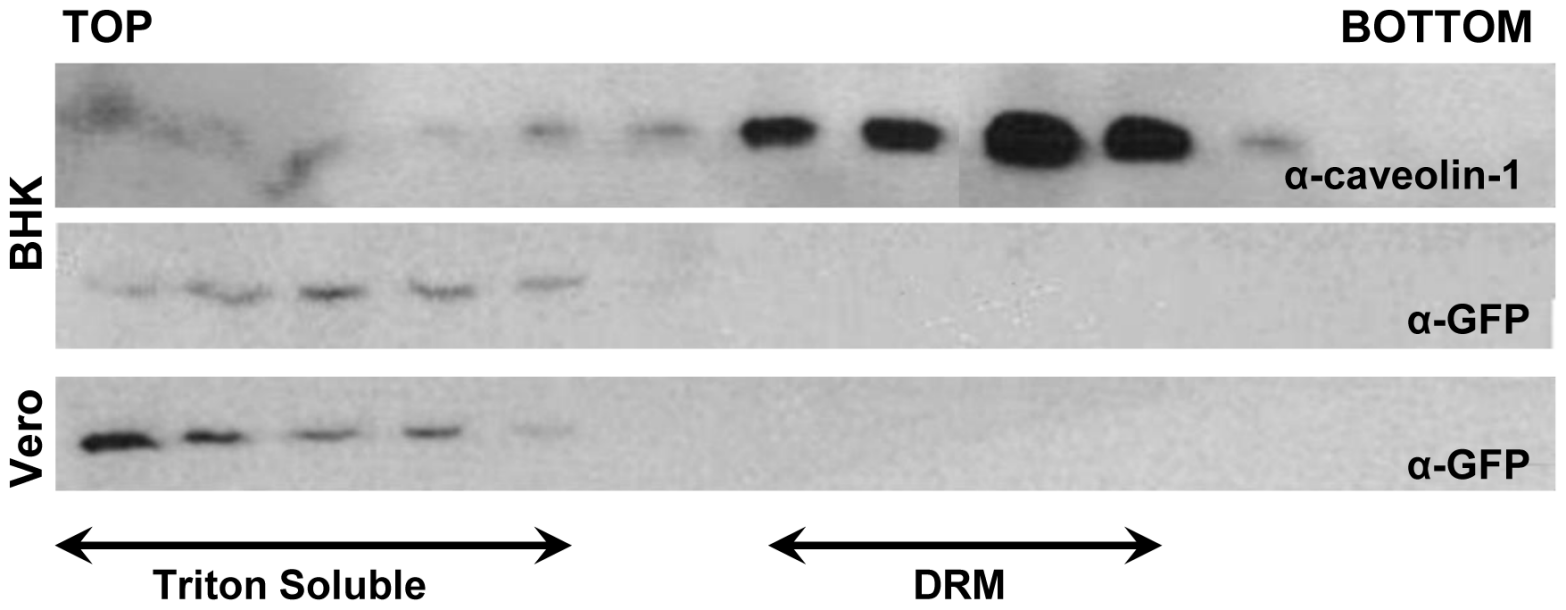
Lack of association of 3A with membranes rich in lipid rafts. Cells were transfected with pEGFP3A and 24 h later lysed with cold 0.5% Triton X-100 in TNE Buffer and the rafts were purified by density gradient fractionation, top and bottom are indicated. Detergent resistant membrane fractions (DRM) are indicated. Proteins in the different fractions were resolved on a 12% SDS-PAGE and blotted with a polyclonal antibody to caveolin-1 and a MoAb to GFP.

Since the previous results showed that 3A protein could be associated with lipid membranes, the possible involvement in this interaction of cholesterol enriched micro domains of lipid rafts was analyzed. The association of different viral proteins to lipid rafts has been described for many viruses [52], [53], [54], [55], [56]. Detergent-resistant membrane (DRM) fractions were isolated by sucrose density gradient from Vero and BHK-21 cells transfected with pEGFP3A (Fig. 6). Western-blot analysis showed no overlapping between the fractions detected by the anti-GFP antibody and those stained with an anti-caveolin 1 antibody (present in lipid rafts), confirming that 3A is not associated with lipid rafts.

### Membrane topology of 3A protein

**Figure 7:**
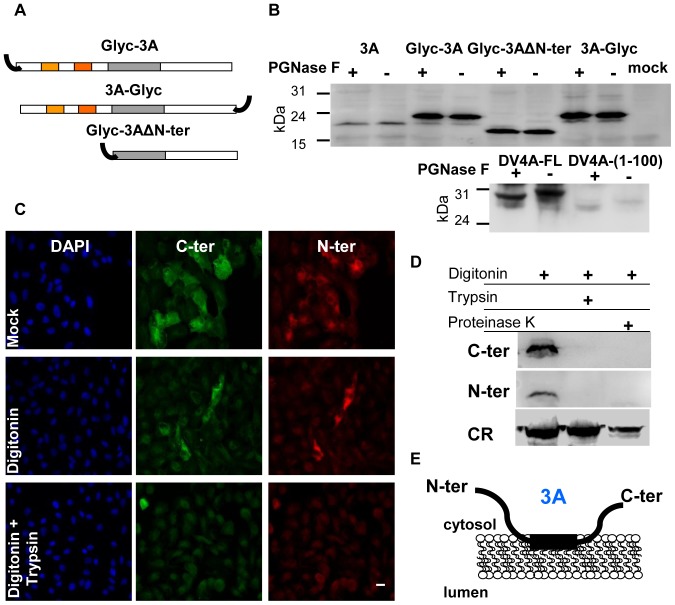
Membrane topology of 3A. A) Schematic representation of fusion proteins of the complete 3A and 3AΔN-ter, with the glycosylation acceptor site Asn-Ser-Thr-Ser-Ala-Asn (black curve lines). For 3A, α-helixes (orange boxes) and the hydrophobic region (gray boxes) are indicated. B) Deglycosylation assay of transiently expressed fusion proteins. Vero Cells were transfected with pcDNAGlyc-3A, 3A-Glyc or Glyc-3AΔN-ter, and 24 h later cells were lysed in NPB and PNGase F treated for 1 h at 37°C. Proteins were separated in 12% SDS-PAGE and blotted with a polyclonal antibody to the C-terminus of 3A (346). As positive control for glycosylation cells previously infected with vaccinia T7/F3 were transfected with pTM-DV4AFL(1–150)-eGFP-Glyc or with pTM-DV4A(1–100)-eGFP-Glyc and processed as before using a MoAb to GFP. C and D) Biochemical protease protection assay of transiently expressed 3A. C) Vero cells grown on coverslips were transfected with pcDNA3A and 24 h later were permeabilized with digitonin for 1 min, treated with trypsin for 5 min and fixed in PFA 4% after proteolysis. Cells were analyzed by immunofluorescence with a MoAb to the C-terminus (2C2) and a polyclonal Ab to the N-terminus (479) of 3A. Alexa fluor 488 anti-mouse and alexa fluor 555 anti-rabbit were used as secondary antibodies. D) Vero cells were transfected and processed as in (C). Cells were lysed and analyzed by Western blotting using polyclonal antibodies to the C- (346) and the N-termini (479) of 3A and to calreticulin (CR). E) Schematic representation of the model proposed for the membrane topology of 3A protein. Cytosol and lumen are indicated. Scale bar, 20 µm.

Characterization of membrane topology of viral proteins contributes to understand the structural organization of viral replication complexes in infected cells. Glycosylation assays can provide information on membrane topology of proteins associated with the ER [57]. To address the membrane topology of FMDV 3A wt, a glycosylation acceptor site was fused in-frame to the C- or N-termini of 3A, as well as to the N-terminus of the HR in a construction with the N-terminus deleted (Fig. 7A). None of the proteins expressed were found glycosylated under the assay conditions used. As expected, glycosylation was observed for dengue virus NS4A protein carrying the same glycosylation acceptor, used as positive control [38] (Fig. 7B), supporting that both 3A protein termini are located towards cytosol.

To confirm the topology suggested by the glycosylation results, a biochemical protease protection assay [58] was performed. Thus, cells transiently expressing 3A were treated with trypsin or proteinase K and analyzed, using antibodies to the N- and the C-termini of the protein, by immunofluorescence (Fig. 7C) as well as by Western blotting (Fig. 7D) [57], [58]. The results revealed that both termini of the protein were proteolyzed indicating that the N- and the C-termini of 3A are accessible to the enzymes and, therefore, oriented towards the cytosol. Indeed, no proteolysis was observed for calreticulin, a protein that resides in the protease-protected ER lumen.

The results obtained led us to propose a membrane topology model (Fig. 7E) in which 3A protein interacts with ER membranes through its hydrophobic stretch, while its N- and C-terminus face the cytosol being accessible to other viral proteins for viral replication.

## Discussion

Replication of positive strand RNA viruses is intimate associated with membranes, which confers advantages not only for viral replication but also in protecting viral RNA from sensing by cell pattern-recognition receptors and the subsequent triggering of innate immunity [59]. The FMDV NS protein 3A is involved in the host range, pathogenicity and virulence of the virus [24], [25], [26], [60] and exhibits properties and characteristics different from those of other picornaviruses. It is thus interesting to gain insight on the function and properties of this “key-protein” involved in FMDV replication.

The picornavirus replication cycle occurs in the cell cytoplasm [61], [62]. Replication complexes appear associated to virus-recruited membrane structures to which NS proteins anchor [63], [64], [65]. In this context, the data available for picornaviruses point to 3A as a multifunctional NS protein [66].

Characterization of protein dynamics in the cell may contribute to understand the different functional roles of viral proteins. To this end, we have *in vivo* studied the properties of GFP fusions with FMDV 3A wt protein and with mutant versions including point mutations that either destabilize dimer formation (L38E and L41E) or impair the establishment of disulfide intermolecular bonds in the odd cysteine residue present in 3A (C65S), as well as deletions corresponding to the N-terminal or the C-terminal regions of 3A protein.

FMDV GFP3A was correctly expressed and its fluorescence displayed a punctuated perinuclear distribution similar to that described for 3A wt in FMDV infected cells [21], [22]. The movements of the GFP3A fluorescent puncta, revealed by time-lapse microscopy, showed a confined track in the cytoplasm. This pattern is different to that associated with microtubules and could be related to the location of the protein in association with the membranes involved in formation of the replication complex. Alterations in the distribution of microtubules and intermediate filaments components have been described in FMDV-infected cells, being 3C(pro) the only FMDV protein involved in these changes [67].

The study by FRAP analysis of the inner dynamics of the sites where GFP3A resides in the cytoplasm revealed that the mobile fraction of the protein was higher at early times pt (70%) and decreased at 24 h pt (about 35%); this later time was chosen for further analyses as it was considered to better reflect the interaction of mature GFP3A with the cell components. Most of the mutations studied, including deletions on the N- and C-termini and the single replacements L41E and C65S, resulted in an increase in protein mobility. Alterations in the mobility and fluorescent pattern of the C-ter deleted mutant could be related with the lack of the interaction domain of 3A with the cellular protein DCTN3 that has been implicated in the motility of viral proteins and whose deletion attenuates the disease in cattle [68]. The lack of effect of replacement 3AL38E on the protein mobility remains to be explained. Taken together, these results indicate that different mutations can alter the interactions responsible for the mobility observed for GFP3A, suggesting a remarkable complexity in the determinants of 3A cellular dynamics.

In FLIP analyses, fluorescence in one area of the cell is repeatedly bleached while images in a non-bleached region are collected. If fluorescent molecules from any other region of the cell can diffuse into the area being bleached, loss of fluorescence will occur in both ROIs, indicating that the regions are connected and the protein can diffuse between them [28]. FLIP experiments have been used to clarify the extent of continuity of various intracellular membrane systems [29], [47]. Here, a monomeric red fluorescent protein targeted to the ER via an immunoglobulin leader sequence and retained in the ER lumen by a KDEL retention signal (IgLdR1kdel) was used as a control of a protein resident in a continuous compartment [32]. When an area in the cytoplasm of cells transiently expressing IgLdR1kdel or GFP3A was repeatedly photobleached, loss of fluorescence was observed to occur in the whole cell cytoplasm, indicating that the protein is located in a continuous compartment. These results, along with the colocalization observed between GFP3A and calreticulin, support an interaction of 3A with the ER, which is consistent with previous data [21], [22].

The interactions of FMDV 3A with cell membranes are poorly understood. In this work we also performed a biochemical characterization GFP3A and of different point and deletions mutants of this protein. In cells transiently expressing 3Awt and GFP3A, these proteins were similarly found in the soluble (about 60%) and the insoluble (about 40%) membrane fractions. The partial association of 3A and GFP3A with cell membranes observed is in agreement with previous analysis of 3A in transfected cells [8]. Interestingly, a fraction of GFP3ABBB was also found in the insoluble fraction indicating that the presence of the 3 copies of 3B does not significantly alter ability for membrane interaction of 3A protein. Deletion of the N-terminus of 3A (GFP3AΔN-ter) significantly increased the solubility of GFP3A. Conversely, deletion of the C-terminus (GFP3AΔC-ter) decreased the solubility, which appeared to be associated to a tendency of this fluorescent protein to accumulate in define points of the cytoplasm (Fig. 4D). On the other hand, an increase in GFP3AΔC-ter mobility was found in FRAP. This apparent discrepancy between solubility and mobility could be due to a bias introduced by the exclusion of cells with highly altered morphology from FRAP analyses.

None of the point mutations analyzed resulted in significant alterations of 3A solubility, suggesting that neither the potential establishment of intermolecular disulfide bridges, nor the efficient 3A dimerization are critical for 3A association to cellular membranes. These results indicate also that residues other than L38 and L41 are likely to be involved in the increase in solubility associated with deletion of the N-terminus of 3A.

Further analyses of the insoluble fraction of GFP3A and GFP3ABBB revealed that high ionic strength and high pH, conditions that favor solubilization of proteins whose binding to membranes mainly depend on electrostatic forces, slightly altered the solubility of GFP3A. The most stringent conditions tested (a chaotropic agent and a non-ionic detergent) enhanced GFP3A solubility, albeit in a non-statistically significant manner. Interestingly, the results obtained with the solubilization treatments demonstrated that GFP3A and GFP3ABBB are not integral membrane proteins, such as calnexin, albeit they can establish strong interactions with intracellular membranes. These results led us to investigate 3A topology by different approaches. The deglycosylation assay used indicated that 3A could display both N- and C-termini towards cytosol, an observation that was confirmed by the protease protection assay. Based on these results, we proposed a model for the 3A membrane topology in which both protein termini are exposed to the cytosol (Fig 7E). This model could be compatible with an infected cell context, where the mature protein and the 3AB precursors would face the cytosol where viral replication takes place and protein-protein interactions expected to occur.

In the model proposed for the interaction of PV 3A/3AB proteins with cell membranes (14), 3A can adopt a transmembrane topology when expressed alone, while its precursor 3AB behaves as a non-transmembrane protein. FMVD 3A differs from the rest of picornaviruses in the length of its C-terminus (66 amino acids longer than PV), which could enable 3A to acquire a cytosolic topology, without the contribution of 3B as required in PV. The non-transmembrane association with intracellular membranes and the display of both protein termini to the cytosol are novel evidences of the differences existing among FMDV 3A and those of other picornaviruses.
